# Analytical Ultracentrifugation as a Matrix-Free Probe for the Study of Kinase Related Cellular and Bacterial Membrane Proteins and Glycans

**DOI:** 10.3390/molecules26196080

**Published:** 2021-10-08

**Authors:** Stephen E. Harding

**Affiliations:** 1 National Centre for Macromolecular Hydrodynamics, School of Biosciences, University of Nottingham, Sutton Bonington LE12 5RD, UK; steve.harding@nottingham.ac.uk; 2 Science for Cultural History (SciCult) Laboratory, Kulturhistorisk Museum, University of Oslo, St. Olavs Plass, 0130 Oslo, Norway

**Keywords:** sedimentation velocity, sedimentation equilibrium, molecular mass, oligomeric state, ligand binding, detergent binding, conformation, SEDFIT-MSTAR

## Abstract

Analytical ultracentrifugation is a versatile approach for analysing the molecular mass, molecular integrity (degradation/aggregation), oligomeric state and association/dissociation constants for self-association, and assay of ligand binding of kinase related membrane proteins and glycans. It has the great property of being matrix free—providing separation and analysis of macromolecular species without the need of a separation matrix or membrane or immobilisation onto a surface. This short review—designed for the non-hydrodynamic expert—examines the potential of modern sedimentation velocity and sedimentation equilibrium and the challenges posed for these molecules particularly those which have significant cytoplasmic or extracellular domains in addition to the transmembrane region. These different regions can generate different optimal requirements in terms of choice of the appropriate solvent (aqueous/detergent). We compare how analytical ultracentrifugation has contributed to our understanding of two kinase related cellular or bacterial protein/glycan systems (i) the membrane erythrocyte band 3 protein system—studied in aqueous and detergent based solvent systems—and (ii) what it has contributed so far to our understanding of the enterococcal VanS, the glycan ligand vancomycin and interactions of vancomycin with mucins from the gastrointestinal tract.

## 1. Introduction

There is growing interest in the structure and interactions of membrane associated proteins and glycans particularly those that are related to kinase activity either as kinases themselves or when phosphorylated by external kinase activity. Examples include the possible influence of Syk kinase phosphorylation on the main function of the polytopic membrane glycoprotein band 3 which is to facilitates the exchange of chloride for bicarbonate in human red blood cells [1,2] and the enterococcal VanS kinase involved in glycopeptide resistance regulation [3,4,5].

The study of such systems—in common with many other membrane-based systems—presents a number of challenges for characterisation:

1. The purification process can lead to critical alterations in the conformation and oligomeric state of the protein/glycoprotein leading to subsequent non-representative structural (oligomeric state/conformation) or interaction information [1,6]
2. Once purified, in common with many membrane-associated proteins and glycoproteins they are usually stubborn non-crystallizers making them not amenable to high resolution crystallographic analysis [7], although low resolution shapes of macromolecules, still considered important [8], are still possible
3. Solubilisation and stabilisation in an appropriate solvent for solution studies (hydrodynamics, NMR) is not easy [9]. (i) For membrane-associated proteins/glycoproteins (integral or peripheral) solubilisation with detergent is required. Different detergents can disrupt the native structure to different degrees, and for interpretation of measurements the average extent of binding (which could be dynamic) of detergent to the protein is normally required for correct interpretation of the data [1,10]; (ii) For proteins/glycoproteins with a significant cytoplasmic domain (or extracellular region) account needs to be made of the different solvent requirements for the cytoplasmic domain (CD) (aqueous [11]) and transmembrane domain (TMD [10]) (non-aqueous or detergent), before appropriate conclusions can be drawn. Claims on oligomeric states based on either detergent based or aqueous based solvent systems alone need to be treated with caution: in such cases separate studies on the CD and TMD domains are useful [10,11]. Addition of further materials to stabilize the structure such as glycerol can also impact on the solution properties [3].
4. Once appropriately solubilised (and stabilised), as before for the TMD domains, the extent of detergent binding is required, taking into account the dynamic nature of the binding process [6]. In addition, the technique(s) chosen to study the oligomeric state and conformation should not disrupt or affect either the intact macromolecule or the CD and TMD domains if being studied individually. This can be difficult with techniques that require a column (e.g., size-exclusion chromatography), separation membrane (e.g., field flow fractionation) or immobilisation onto a surface (atomic force microscopy, surface plasmon resonance).

One technique which is gaining increased popularity for the characterisation of membrane based and other systems is the analytical ultracentrifuge [1,3,4,5,10,11,12,13,14,15,16,17,18,19,20,21]. This instrument is matrix free in the sense that it does not require a separation column or membrane with possible non-inertness complications but is based on the creation and analysis of concentration distribution of macromolecules in solution under the influence of a centrifugal field. It provides information about the molecular integrity of the molecule (presence/absence of dissociation products, aggregates), the molecular mass and oligomeric state, the strength of interactions (either associative or complexes with other molecules) and low-resolution conformation information in terms of overall shapes of molecules [21].

In this short review we will consider the two main analytical ultracentrifuge techniques—sedimentation velocity and sedimentation equilibrium—and the supportive methodology required. Our principal example will be the cellular band 3 glycoprotein system [1,10,11]. Although that work was done over two decades ago it is relevant to ongoing studies on bacterial membrane kinase systems [2,22,23,24]. We will also consider some of the studies done so far on the enterococcal kinase VanS system under aqueous only conditions together with the interactions involving the ligand vancomycin. Relevant to oral-based formulations we will also consider how the analytical ultracentrifuge has revealed strong interactions between vancomycin and mucin [25]. To avoid the review becoming opaque to the non-specialist without a background in hydrodynamics, a minimum of methodological detail is given, but key follow-up references are provided instead.

## 2. Analytical Ultracentrifugation: What It Can Tell Us

Analytical Ultracentrifugation is high speed ultracentrifugation—the high speeds needed to produce sufficient movement or redistribution of a macromolecular component in solution—with an in-built optical system to provide an analytical record of this movement or redistribution [21,26,27]. It is not a new technique –the invention was made a century ago by T. Svedberg and colleagues at the University of Uppsala [21]. However, the last 3–4 decades have seen a steady growth in interest with advances in data capture and software making the technique available to a wide range of researchers [21,28] and not just the preserve of a few specialists. However, it is probably fair to say for advanced applications it remains as the provenance of expert users.

It is a powerful tool for assessing the macromolecular integrity (presence and effect of impurities, degradation or aggregation products), the state of oligomerization of a macromolecule, and also self-association reactions [21,29] as well as assaying protein–ligand interactions [30] without the need for immobilization, labelling or an assumed inert separation matrix. It can also yield conformation information: not at high-resolution such as X-ray crystallography or nuclear magnetic resonance, nor even secondary structure information that circular dichroism can provide but at the overall gross conformational level (ellipsoids and bead models), particularly when used in conjunction with data from other techniques [31]. With membrane systems, if detergents are required for solubilization, knowledge of the extent of binding of the detergent to the protein is needed (from, for example, ^14^C measurements [6,32] and MALDI-TOF [33]) as well as the partial specific volume $\bar{v}\;$of the protein or glycan (this can be obtained from density measurements—see [29,34]), essential for buoyancy effects to be taken into account.

## 3. Sedimentation Velocity Analytical Ultracentrifugation

**Figure 1:**
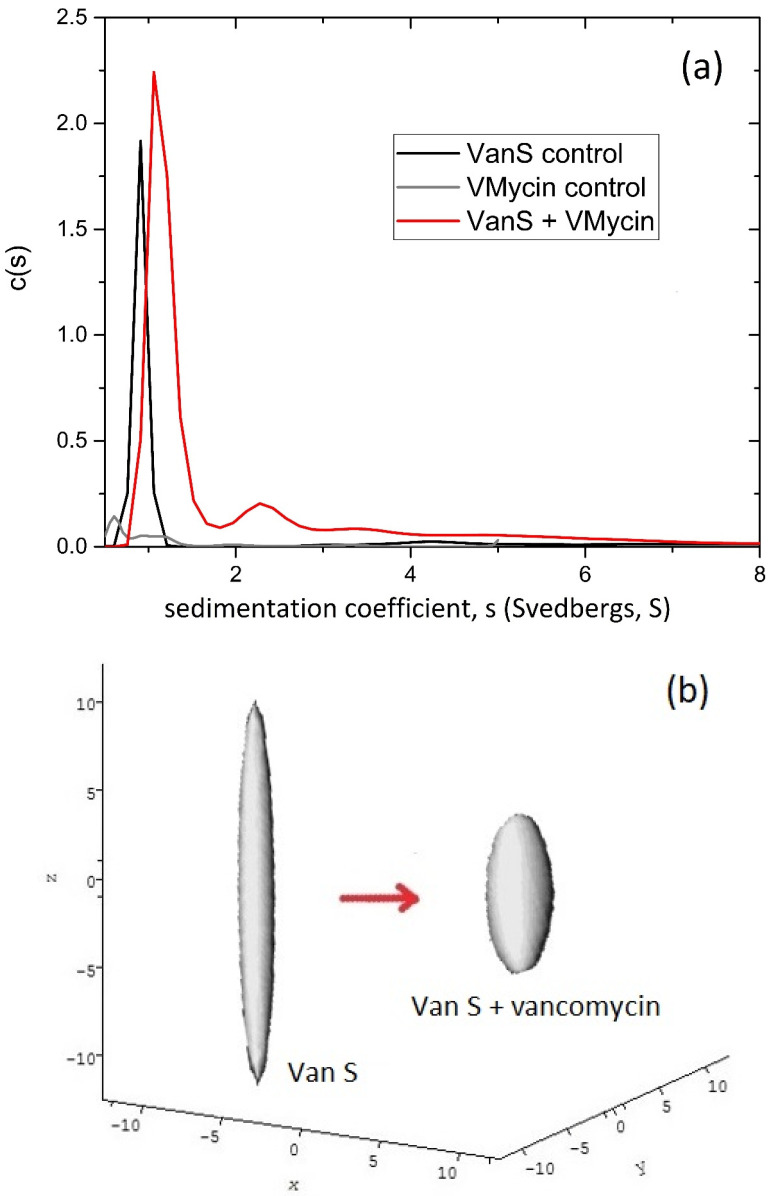
Hydrodynamics of enterococcal VanS (**a**) Sedimentation coefficient concentration distribution, c(*s*) vs. *s* profile from SEDFIT analysis [35] for VanS (black profile) in aqueous buffer pH ~7.9, *I* = 0.1 M (supplemented with 20% glycerol) at 20.0 °C and a loading concentration of 0.25 mg/mL (5.4 μM). Additionally, shown is the profile for vancomycin 0.019 mg/mL (12.8 μM) (grey profile) and a mixture of VanS and vancomycin (red profile) under the same conditions. (**b**) Hydrodynamic shape (equivalent prolate ellipsoid) of the enterococcal VanS protein from ELLIPS1 [31] in the absence (left) and presence (right) of vancomycin. The shift in the sedimentation coefficient is equivalent to a reduction in axial ratio of the equivalent prolate hydrodynamic ellipsoid from ~12:1 to a more compact structure of axial ratio ~5:1. Adapted from Phillips-Jones et al. [3] and reproduced by permission from the Nature Publishing Group.

Sedimentation velocity experiments are run at relatively high speeds (up to 60,000 rev/min or ~200,000 g) resulting in sedimentation of the macromolecular solute: the change in the radial concentration distribution of solute with time is measured to yield the (weight average) sedimentation coefficient, *s* (=the ratio of the sedimentation rate to the centrifugal field) as well as the distribution of sedimentation coefficients [21,26]. A popular algorithm for performing the transformation is SEDFIT [35] which provides not only *s* but also the sedimentation coefficient distribution—g(*s*) versus *s*—important for assessing the molecular integrity/purity of the system (and this is why it is regarded by the Food and Drug Adminstration, FDA as a gold standard method for assessing the molecular integrity (absence of degradation products or aggregates) in monoclonal antibody based formulations from BioPharma. Since diffusive effects can broaden the peaks representing each component, SEDFIT can also provide a distribution corrected for diffusion broadening, c(*s*) versus *s*. Figure 1a gives an example of such a diffusion corrected c(*s*) distribution for the enterococcocal VanS kinase in aqueous buffer pH ~7.9, ionic strength, *I* = 0.1 M (supplemented with 20% glycerol), which shows a significant shift (at the same concentration) in the presence of the low molecular mass glycopeptide ligand vancomycin [3].

Although sedimentation coefficient distributions depend on conformation as well as molar mass (in g/mol, or equivalently ‘molecular mass’—which we use here—or ‘molecular weight’ in Da), it is possible to transform distributions to molecular mass distributions for monodisperse solutions or mixtures with a small number of components with SEDFIT. For more polydisperse systems—including those with a quasi-continuous distribution of sizes—the transformation is still possible using additional information from sedimentation equilibrium or light scattering, in what is known as the *Extended Fujita* method [36], and this has also been incorporated with the SEDFIT suite of algorithms.

## 4. Sedimentation Equilibrium Analytical Ultracentrifugation

In sedimentation equilibrium [37] the ultracentrifuge is run at lower rotational speeds so that the back forces due to diffusion become comparable with the centrifugal forces. At equilibrium, frictional effects and hence conformational effects are eliminated and the optical records give a direct or *absolute* measure of molecular mass/oligomeric states and association constants, again at a range of concentrations [29].

The primary information is the weight average molecular mass, *M*_w_. From the way *M*_w_ changes with loading concentration, or the way *M*_w_(*r*) versus *c*(*r*) varies within a run—or by modelling the *c*(*r*) versus radial displacement distributions directly—it is possible to ascertain the stoichiometry and reversibility of self-associative or heterologous interactions.

Popular algorithms are SEDFIT-MSTAR [37] for determining average molecular masses, MULTISIG [38] for distributions and SEDPHAT [39] for analysis of interacting systems. Non-ideality effects are taken care of by working at low concentration (<0.5 mg/mL), estimation and incorporation of the non-ideality second virial coefficient [40,41,42]; or extrapolation to zero concentration [27]. The easy-to-use SEDFIT-MSTAR algorithm is based on the M* function [43]—designed for the analysis of difficult heterogeneous systems (of which membrane kinases belong to) and built into the FORTRAN program MSTAR [44] then BASIC [45] and finally incorporated into the SEDFIT suite of algorithms based on C + [37].

## 5. Band 3 Protein

One of the most extensively studied kinase related membrane systems by the analytical ultracentrifuge is the human erythrocyte anion transporter protein known as band 3 [1,6,11,22]. Band 3 is the most abundant protein/glycoprotein in red blood cells and facilitates the one-to-one exchange of chloride for bicarbonate [46]. When subject to oxidative stress red blood cells respond by activating tyrosine kinases determining the tyrosine phosphorylation of band 3, which is thought to regulate its own phosphorylation [2]. The band 3 protein/glycoprotein itself is also one of the few membrane-related kinase systems whose oligomeric state and conformation has been thoroughly studied using hydrodynamic/analytical ultracentrifuge techniques. We now review that earlier work and consider its impact for ongoing studies on bacterial kinase systems such as the VanS system and its interactions with the antibiotic vancomycin.

**Figure 2:**
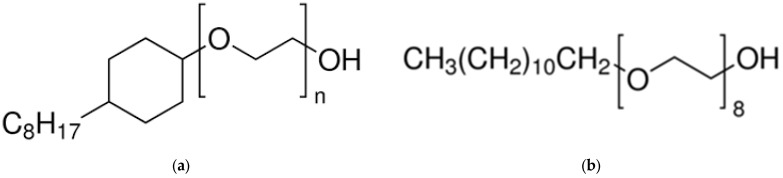
(**a**) Reduced tritonX-100 and (**b**) octaethylene glycol monododecyl ether (C_12_E_8_). Courtesy Sigma-Aldrich limited.

Sedimentation equilibrium and sedimentation velocity was used to assess the molecular integrity, oligomeric state and conformation of band 3 after appropriate solubilisation, and the results summarised in Table 1. The intact band 3 protein was solublised in the detergent reduced tritonX-100 (Sigma-Aldrich)—Figure 2a (reduced triton was chosen because of its uv invisibility allowing optical registration of sedimentation profiles of the protein using uv absorption optics in addition to Rayleigh interfence). Experiments on the isolated trans-membrane domain (TMD) were performed in the detergent octaethylene glycol monododecyl ether C_12_E_8_ (Sigma-Aldrich). Band 3 also has a significant cytoplasmic domain (CD) which normally exists in an aqueous environment. A comparative study was conducted, comparing the properties of the intact band 3 and the isolated TMD domain in the appropriate detergents with the properties of the isolated CD domain in aqueous solvent (pH8.0 Tris-HCl buffer containing 10 mM Tris HCL, 10 mM NaCl and 0.5 M EDTA).

### 5.1. Estimation of Detergent Bound

The first step was to estimate the amount of detergent bound to the intact band 3 protein and TMD so as molecular mass estimates for the protein could then be corrected. The method of Casey and Reithmeyer was employed [6]. This is a chromatographic method involving tracer ^14^C labelled detergent and assaying for radioactivity and protein content, correcting for background levels.

### 5.2. Partial Specific Volume $\bar{v}$

This is required for evaluation in the buoyancy term in the equations for sedimentation velocity and equilibrium. This can be calculated from the amino acid composition and (for intact band 3 and the TMD) from the known amount of detergent bound using a method outlined by, e.g., Durchslag and Zipper [47]. Alternatively, it can be measured using a mechanical oscillator-based density meter [48].

### 5.3. Weight Average Molecular Mass

**Figure 3:**
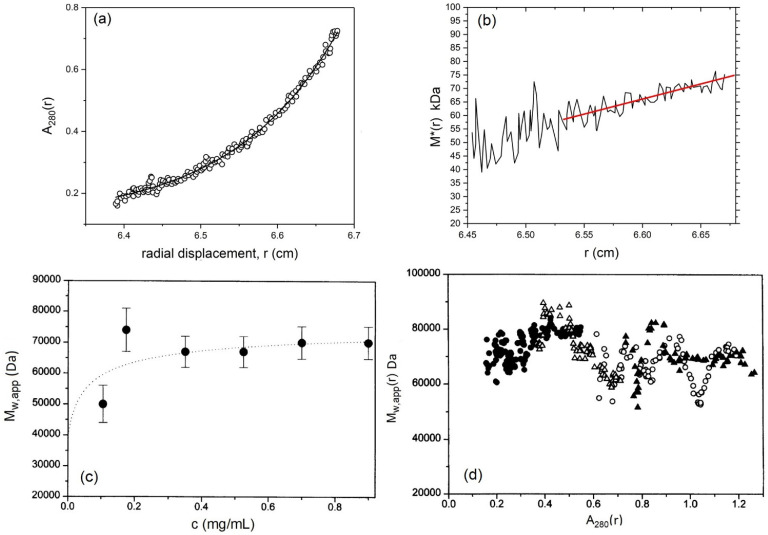
Sedimentation equilibrium characterisation of the cytoplasmic domain of the band 3 transporter protein. (**a**) Concentration distribution (expressed in terms of UV absorbance at 280 nm in a 12 mm optical path length cell) versus the radial displacement at a given position in the cell. Equilibrium speed 10,000 rpm (~8000 g, depending on radial position), temperature 20.0 °C, loading concentration *c* = 0.18 mg/mL. The line fitted is for an ideal reversible dimerization with a molar dissociation constant *K*_d_ = (2.8 ± 0.5) μM (**b**) Obtaining the apparent weight average molecular mass *M*_w,app_ for the whole distribution of macromolecular components. The integral function *M* * [43] is used which yields *M*_w,app_ over the whole distribution when *r* reaches the cell base position (indicated by the red line extrapolation). Same conditions is in (**a**). At low loading concentrations (such as the case here) *M*_w,app_ = *M*_w_, the thermodynamically ideal weight average molecular mass. (**c**) Plot of apparent weight average molecular mass versus cell loading concentration, *c*. The dotted curve is a fit for an ideal reversible dimerisation with dissociation constant, *K*_d_ ~3 μM, in agreement with (**a**). (**d**) “Point” apparent weight average molecular masses *M*_w,app_(*r*) at individual points or radial positions in the ultracentrifuge cell plotted against local concentration *A*(*r*) in absorbance units at 280nm for different loading concentrations (different symbols). Adapted from Cölfen et al. [11] and reproduced by permission of The Biophysical Society.

Next the weight average molecular mass can be measured using the MSTAR procedure (now popularly incorporated into the SEDFIT suite of algorithms as SEDFIT-MSTAR [37]). Figure 3 shows analysis of the CD domain in 10 mM tris-HCl buffer (pH8.0) supplemented with 10 mM NaCl and 0.5 mM EDTA, and at a protein loading concentration of 0.18 mg/mL. Figure 3a shows the concentration distribution c(*r*) vs. *r* at sedimentation equilibrium and Figure 3b shows the MSTAR extraction of the weight average molecular mass *M*_w_ of all the macromolecular species in solution in the ultracentrifuge cell. A cumulative integral function M*(*r*) [43] works along the *c*(*r*) vs. *r* curve until the cell base is reached, where M* = *M*_w_ for the whole distribution (Figure 3b). The value for *M*_w_ = 80,000 Da obtained corresponds to a dimer species. The existence of mostly dimer is confirmed by fitting the concentration distribution of Figure 3a to a monomer-dimer model which yields a very low dissociation constant *K*_d_ = (2.8 ± 0.5) μM, consistent with a strong dimerisation. The experiment can be repeated at a number of different initial loading concentrations, *c*, and Figure 3c shows a plot of *M*_w_ vs. *c*, showing dimer which eventually noticeably dissociates into monomer at concentrations <0.1 mg/mL. Finally, as a check for reversibility of the dissociation estimates from the change in the local concentration in the ultracentrifuge cell *c*(*r*) with radial position *r* can be made of the local or point average weight average molecular mass *M*_w_(*r*) and how it changes with *r* or *c*(*r*) from a given run at particular initial loading concentration, *c*. Because this involves differentiations along the c(*r*) vs. *r* (or ln*c*(*r*) vs. *r*^2^) curves, the data become noisier. However, despite the extra noise (Figure 3d) the different data sets of *M*_w_(*r*) vs. *c*(*r*) for different loading concentrations, *c*, all seem to overlay– symptomatic of a reversible dimerisation [49,50].

### 5.4. Sedimentation Coefficient and Low Resolution Conformation

**Figure 4:**
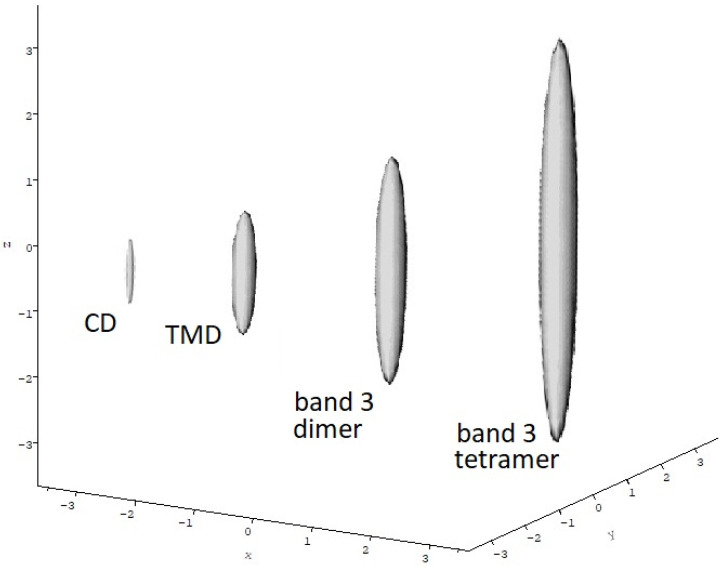
Comparative hydrodynamic shapes for band 3 cytoplasmic domain dimers, transmembrane domain dimers, intact band 3 dimers and intact band 3 tetramers. ELLIPS1 routine used based on the Perrin frictional shape parameter *P*. Solvent conditions as in Table 1. Shapes scaled according to molecular mass.

Sedimentation velocity profiles revealed a single sedimenting boundary species, consistent with almost pure dimer *s*^o^_20,w_ = (3.74 ± 0.07) S. Combination of this with the molecular mass (for the dimer) yielded a value of 1.7 ± 0.2 for the translational frictional ratio *f/f_o_* where *f* is the frictional coefficient of the macromolecule and *f_o_* is the frictional coefficient for a spherical particle of the same molecular mass and anhydrous volume [31,51]. For a spherical anhydrous particle *f/f_o_* = 1. If the macromolecule is either hydrated (has significant solvent dynamically associated with it) or asymmetric (or both) then the frictional ratio wll be >1. After a correction for hydration an estimate for the “frictional ratio due to shape” or Perrin frictional shape function *p* of ~1.5 is obtained which corresponds to a prolate ellipsoidal particle of axial ratio *a/b* ~10:1 using the routine ELLIPS1 from the ELLIPS suite of macromolecular shape algorithms [31,51] (Figure 4).

Table 1 compares the properties of the isolated TMD domain and intact band 3 molecule with this. By contrast to the CD, the transmembrane domain is a stable dimer with no sign of dissociation and has a more spheroidal conformation in solution (*a*/*b*∼3.5:1), consistent with 2D crystal images from electron microscopy. Again, by contrast the intact band 3 protein showed a more complex dimer–tetramer equilibrium with evidence of hexamer (depending on preparation protocol) and some higher order associations [10]. Figure 4 compares the relative sizes and overall shapes (in terms of hydrodynamic prolate ellipsoids) of the CD dimers, TMD dimers and the dimer and tetrameric forms of the intact band 3. One can also clearly see why, despite the cytoplasmic domain, the intact band 3 protein is soluble in the detergent: the TMD dominates the molecule.

Parallel studies of the protein and its aqueous and detergent soluble domains have thus provided a useful insight into the olgomeric state and conformational properties of this important protein and have provided the basis for further investigation of the changes that occur after irreversible oxidation and phosphorylation after oxidative stress, and potential self-regulation of its kinase/phosphorylation [2]. Furthermore, although not bacterial the hydrodynamics of band 3 are well understood and give a good model for which bacterial kinase systems can be based—such as the VanS system.

## 6. Enterococcal VanS—Vancomycin System

The extensive work on band 3 provides the basis for comparison for a current ongoing hydrodynamic study on the enterococcal VanS kinase membrane protein (of monomer molecular mass M_1_~47 kDa) and its interaction with the aqueous soluble antibiotic glycan vancomycin (M_1_ = 1449 Da) [3,4,5]. The importance of the enterococcal VanS kinase system and relevance to antimicrobial resistance has been considered elsewhere in this volume by Ma and Phillips-Jones [52]. Our focus has been on the hydrodynamic properties of the VanS system in the presence and absence of vancomycin, as well as the self-associative properties of vancomycin and its complexation with mucin glycoproteins of relevance to the oral administration of this drug which is mainly administered intravenously. Although the hydrodynamic work on VanS has thus far only considered the properties in aqueous solvents where the protein has limited solubility some interesting properties have been observed.

Like band 3, enterococcal VanS has a transmembrane region (two domains) but by proportion a much larger cytoplasmic region, together with an extracellular domain and unlike band 3 this renders a limited but nonetheless significant aqueous solubility for the intact molecule. As a first part in the characterisation process VanS was therefore characterized in aqueous solution (10 mM HEPES = 100 mM NaCl, pH 7.9, ionic strength, I = 0.1 M supplemented by 20% glycerol and run at 7.0 °C to maintain stability) by sedimentation velocity and sedimentation equilibrium in the analytical ultracentrifuge. Under these solution conditions [3,4] enterococcal VanS was surprisingly found to be monomeric by sedimentation equilibrium as analysed by both SEDFIT-MSTAR [37] and MULTISIG [38] which yielded a weight average molecular mass *M*_w_ of (47 ± 1) kDa, with some evidence of a small amount (~1%) of tetramer. The sedimentation coefficient distribution (Figure 1a) obtained using SEDFIT [35] showed primarily a single species with a small amount of higher molecular mass species, presumably tetramer. Combination of the sedimentation coefficient, *s* = (0.9 ± 0.1)S (corresponding to a value for *s*_20,w_ = 2.3 S after normalization to the density and viscosity of water at 20.0 °C) and molecular mass of the monomer, gave a high value for the Perrin translational frictional function *p* = (1.64 ± 0.09), which yielded, using the routine ELLIPS1 [31,51] a (prolate) axial ratio of ~(12 ± 2). In the presence of vancomycin—whose own sedimentation coefficient is <0.5 S—VanS experienced a significant shift of the sedimentation coefficient by >30% from *s* = (0.9 ± 0.1) S to *s* = (1.2 ± 0.2) S (corresponding to a shift of *s*_20,w_ = from 2.3 to 3.1 S). An identical protein concentration was used to ensure there were no complications through different concentration dependent effects. Since in addition there was no change to the molecular mass from sedimentation equilibrium in the same solvent conditions—as shown by SEDFIT-MSTAR analysis [3,4]—this indicated that the change in *s* (or *s*_20,w_) is most likely due to a change to a more compact conformation (Figure 1b).

However, further investigations of both band 3 and VanS membrane proteins are likely to be revealing especially in detergent solubilised conditions. For this a new method for the assay of detergent binding based on matrix-assisted laser desorption/ionization mass spectrometry (MALDI-TOF MS) now allows quantification of pure or mixed detergents in complex with membrane proteins and without the need for radioisotopes [33]. In cases where it is not possible to apply the radioisotope or MALDI-TOF methods, in principle the degree of detergent binding could be included as an extra fitting parameter in the analysis of the sedimentation records [16], but this requires the use of both the uv-absorption (within Lambert-Beer law restrictions) and Rayleigh interference optics and results can be obscurred by the presence of micelles: the MALDI-TOF MS method would therefore be the current method of choice.

### Vancomycin Dimerisation

**Figure 5:**
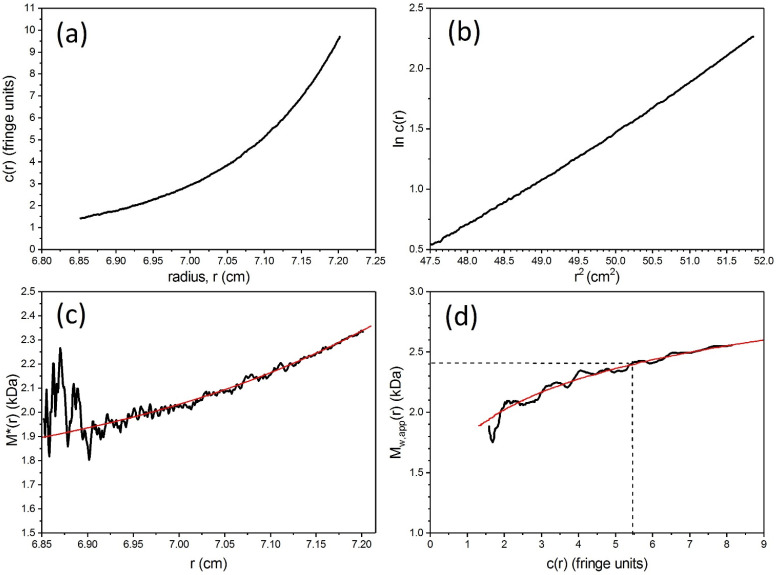
Sedimentation equilibrium analysis using SEDFIT-MSTAR for vancomycin. Solvent 0.9% NaCl at 7.0 °C, at a loading concentration *c* of ~1.25 mg/mL. (**a**) Concentration distribution *c*(*r*) (expressed in terms Rayleigh interference displacement units in a 12 mm optical path length cell) versus the radial displacement *r* at a given position in the cell (**b**) corresponding plot of lnc(*r*) vs. *r*^2^. Departure from linearity is consistent with self-associative behaviour. (**c**) extrapolation of the *M* * integral function to the cell base to yield the whole distribution weight average molecular mass *M*_w,app_ = (2.4 ± 0.1) kDa (**d**) plot of the local or point average molecular mass *M*_w,app_(*r*) as a function of local concentration *c*(*r*) in the ultracentrifuge cell obtained by taking a derivative of the data from plot (**b**). The dashed line gives the value of *M*_w,app_(*r*) at the “hinge point”, i.e., at the value of *c*(*r*) which equals the loading concentration. This also = *M*_w,app_, the (apparent) weight average molecular mass for the whole distribution and gives a check on the value obtained from (**c**). For other solvent conditions see [4]. Adapted from Phillips-Jones et al. [4] and reproduced by permission from the Nature Publishing Group.

**Figure 6:**
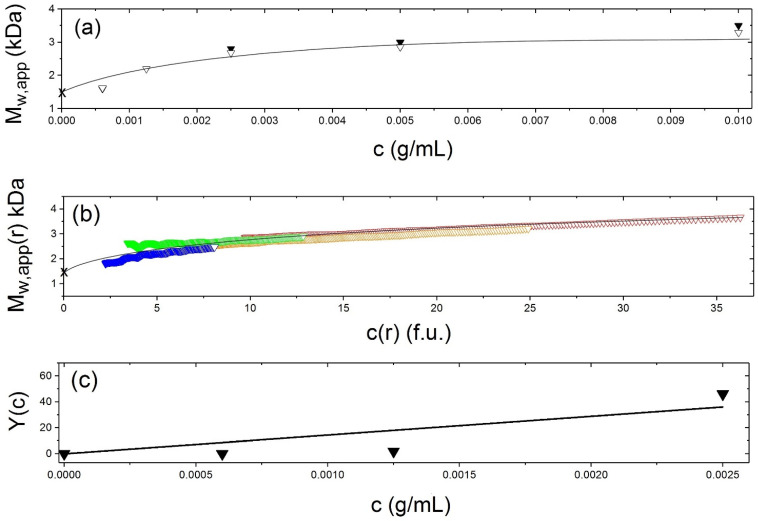
Sedimentation equilibrium dimerisation analysis of vancomycin. Solvent 0.9% NaCl at 7.0 °C. using (**a**) Change of weight average molecular mass *M*_w_ of vancomycin with loading concentration. Solid triangle—molecular masses *M*_w,app_ obtained from *M* * analysis using SEDFIT-MSTAR [37]. Open triangles—molecular masses obtained by hinge point analysis also using SEDFIT-MSTAR. (**b**) Diagnostic plots confirming a completely reversible dimerisation. Weight average molecular masses *M*_w_(*r*) at individual radial positions in the ultracentrifuge cell plotted against local concentration *c*(*r*) in interference fringe units for different loading concentrations: blue (1.25 mg/mL), green (2.5 mg/mL), orange (5.0 mg/mL) and red (10.0 mg/mL). For a completely reversible association the plots should lie, within experimental error, on the same curve. (**c**) Evaluation of the association constant *k*_2_ and corresponding molar dissociation constant *K*_d_ and standard Gibbs free energy change *G*^o^ from the Kegeles-Rao equation [21,53,54]: *Y*(*c*) ≡ *M*_1_{*M*_w_(*c*) − *M*_1_}/{(2*M*_1_ − *M*_w_(*c*))^2^} = *k*_2_.*c*, where the *M*_w_(*c*) are the weight average molecular masses (averaged over whole macromolecular distributions) at different loading concentrations, *c*. *k*_2_ = (14,400 ± 3600) mL/g, *K*_d_ = (40 ± 10) μM and *G*^o^ = (23.3 ± 0.6) kJ/mol. Because of the low molecular masses non-ideality effects can be assumed negligible and *M*_w_,_app_ = *M*_w_ in (**a**–**c**). For corresponding analyses in other solvent conditions see [4]. Adapted from Phillips-Jones et al. [4] and reproduced by permission from the Nature Publishing Group.

The dimerization equilibrium behaviour of vancomycin has also recently been examined [4], again using SEDFIT-MSTAR, with a range of solvent conditions and loading concentrations. As we did with the CD domain of band 3 [11], diagnostic plots of the weight average molecular mass values *M*_w_(*r*) at individual radial positions in the ultracentrifuge cell were generated, in this case for each of four relevant solvent conditions, namely 10 mM HEPES; 10 mM HEPES + 100 mM NaCl; 10 mM HEPES + 100 mM NaCl + 20% glycerol; and finally 0.9% (*w*/*v*) NaCl in deionised, distilled water (see, e.g., Figure 5—all plots can be seen in Phillips et al. [4]. Plots of the weight average molecular mass versus loading concentration were also produced and an example is shown in Figure 6a for vancomycin in 0.9% NaCl in deionised distilled water. As with the band 3 CD the diagnostic test of overlap of point weight average molecular masses *M*_w_(*r*) vs. local concentration in the centrifuge cell *c*(*r*), where *r* is the radial position, were also plotted for different loading concentrations confirmed a fully reversible dimerization process, with some evidence of further association (Figure 6b). Classical Kegeles and Rao [53] analysis (see also ref [54]) of the sedimentation equilibrium data showed that unlike the strong dimerisation of the cytoplasmic domain of band 3 the vancomycin dimerization was a relatively weak one, with molar dissociation constants ranging from 35 to 50 μM across the range of solvent conditions, again we show the example for 0.9% NaCl (Figure 6c) as solvent but plots for the other 3 solvent conditions can be found in ref [4]. As a direct consequence of this, one of the important finds of this study was that at the clinical infusion concentration of 5 mg/mL the vancomycin glycopeptide was completely dimerized whilst at a concentration of 19 µg/mL—which represents a clinical target “trough” serum concentration, i.e., the target concentration of drug in the blood immediately before the next dose is administered by the clinician/patient—the vancomycin was <20% dimerized, i.e., mostly monomeric [4]. This information is very important to the clinician who has to make sure there is enough dose for the drug to take effect without overdosing because of harmful side-effects. Hydrodynamic studies like this can therefore provide a key guide.

This hydrodynamic approach may have more widespread relevance and a follow up study is currently underway to compare this behaviour with other glycopeptide antibiotics such as teicoplanin [55].

## 7. Vancomycin-Mucin Interactions

The antibiotic vancomycin is normally administered intravenously but can also be administered orally to treat bacterial based gasterointestinal diseases such as pseudomembranous enterocolitis. One of the issues however that are peculiar to oral administration is the possible complications through potential interactions with gasterointestinal mucus and in particular the mucin glycoprotein component: A study involving analytical ultracentrifugation reinforced by quasi-elastic or “dynamic” light scattering (DLS) and environmental scanning electron microscopy (ESEM) was therefore undertaken to explore for the presence of interactions which could help explain the poor absorption of the antibiotic from the intestine and the glycopeptide impacts on the intestinal microbiota and its connection with antimicrobial resistance [25]. A somewhat different AUC approach to probe these large supramolecular aggregation reactions rather than the study of the well-defined dimerisation (vancomycin by itself) or dimerisation/tetramerisation (band 3) described above, was taken.

For the investigation of large aggregation reactions such as those involving vancomycin with mucin the powerful molecular hydrodynamic assay of *co-sedimentation* in the analytical ultracentrifuge has proven useful [30]. High rotor speeds (~45,000 rpm or 130,000 g for a standard Beckman XL-I rotor) are employed to sediment mucin in solution whilst low rotor speeds (~3000 rpm) are used to sediment large supramolecular complexes of molecular mass >10^8^ Da. We can also monitor for the loss of material from the solution through aggregation using this procedure.

Three concentrations of vancomycin were used (i) 0.125 mg/mL where the vancomycin is mostly monomeric (ii) at 1.25 mg/mL (a typical concentration found in stools of patients given oral vancomycin [25]) it is approximately 50% dimerized and (iii) at 12.5 mg/mL where it is mostly dimeric. Concentrations of mucin were chosen to be low enough to be in the dilute region (non-molecular overlap): 0.5 mg/mL for gastric mucin, 0.5 mg/mL for intestinal mucin and 1.0 mg/mL for the smaller submaxillary mucin.

### Co-Sedimentation Assay for Mucin-Vancomycin Complexation

**Figure 7:**
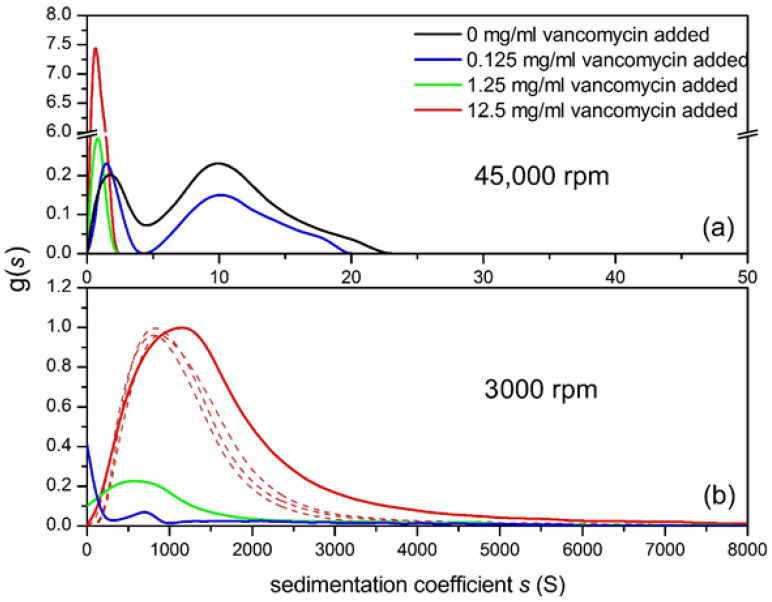
Sedimentation coefficient distribution of pig gastric mucin (PGM)/vancomycin mixtures at different mixing ratio (**a**) at 45,000 rpm (**b**) at 3000 rpm, 0.5 mg/mL PGM + 0.125 mg/mL (blue line), +1.25 mg/mL (dark green), +12.5 mg/mL (red) vancomycin. The 0.5 mg/mL PGM control is shown in black. The dashed lines represent repeats for 12.5 mg/mL vancomycin added. Solvent: 0.1 M PBS (pH 7.0). For further examples see [25]. Adapted from Dinu et al. [25] and reproduced by permission from the Nature Publishing Group.

The 45,000 rpm plot (Figure 7a), shows the 0.5 mg/mL gastric mucin control (no vancomycin added) revealing two components with the main macromolecular mucin component of *s* values between 5 and 23 S and what happens to this as the vancomycin concentration is increased to 12.5 mg/mL. The amount of this component diminishes dramatically through complexation as the vancomycin concentration is increased. The 1.25 and 12.5 mg/mL additions indicate complete interaction of the mucin but this would be expected as the mucin concentration is much lower than the concentration of vancomycin. At 3000 rpm (Figure 7b), there is further insight as the addition of vancomycin has clearly produced large aggregates, (~1500 S), increasing as the vancomycin concentration is increased, leaving little macromolecular mucin behind as the vancomycin concentration is progressively raised.

**Figure 8:**
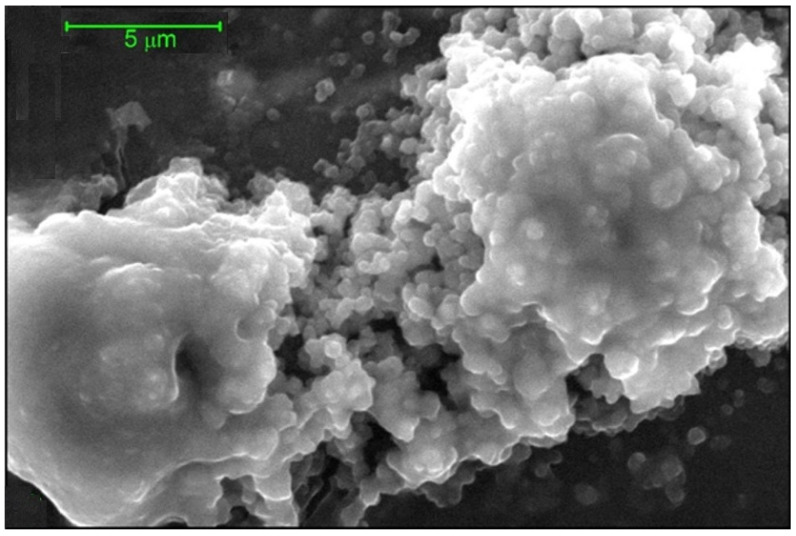
Environmental scanning electron micrograph of vancomycin –pig gastric mucin complex suspended in 0.1 M PBS (pH 7.0). Aqueous samples were subjected to dehydration in the ESEM sample chamber at operating pressures ranging from ~4 to ~5 Torr. For further examples see [25]. Adapted from Dinu et al. [25] and reproduced by permission from the Nature Publishing Group.

Similar behaviour was seen for interactions of vancomycin with intestinal mucin and submaxillary mucin: unequivocally large complexes are formed, which were also detected by DLS and visualized by ESEM, and an example of the latter for the vancomycin-gastric mucin complex is shown in Figure 8.

These measurements have all clearly demonstrated strong complexation between vancomycin and model mucins from different parts of the GI tract. The strongest interactions—with very large complexes being formed appear to be associated with mucus originating from the stomach and small intestine, compared with the mouth. The lower degree of association of the latter may be connected with the lower degree of glycosylation of submaxillary mucins, that is to say it is the carbohydrate region of mucins that is largely responsible for the strong complexation with vancomycin. To test this further, similar studies could be done on mucins that have been O-deglycosylated to different degrees. It is also worth pointing out that we have followed the common practice of using animal mucus/mucins as models for human mucin, and the interactions of mucins from human sources need to be explored as and when the latter become more readily available. The possible interactions of other orally administered antibiotics need also to be explored—for example another glycopeptide teicoplanin used as an alternative to vancomycin for the treatment of pseudomembranous colitis and *Clostridium difficile*—associated diarrhoea [55].

The demonstration of complexation/depletion interactions for model mucin systems with vancomycin also provides the basis for further study on the implications of complexation on glycopeptide transit in humans, antibiotic bioavailability for target inhibition, in situ generation of resistance and future development strategies for absorption of this antibiotic across the mucus barrier.

## 8. Conclusions

It is hoped that this short but targeted review has provided a snapshot of the usefulness of the analytical ultracentrifuge for the study of membrane associated proteins and glycans associated with kinase activity, adding to the powerful array of techniques now available and covered in recent reviews by Phillips-Jones and colleagues [52,56]. We have focused on two examples—one for a membrane protein/glycan from a human cellular system—the band 3 protein which has been fully studied in aqueous and detergent systems reflecting the cytoplasmic and transmembrane domains, respectively. The other the enterococcal bacterial VanS system and the associated antibiotic glycan ligand vancomycin.

We hope also we have been able to demonstrate the considerable versatility of the analytical ultracentrifuge method through its two main variants—sedimentation velocity and sedimentation equilibrium for the study of the oligomeric state, conformation and interactions and under both aqueous and membrane-like detergent environments. Not only are these analytical ultracentrifuge methods particularly useful because they do not require separation media, columns or membranes or immobilisation onto a surface, they can in addition form a seminal part of a powerful array of biophysical techniques as we have seen for the vancomycin-mucin systems. Although perhaps insufficiently considered in the past the analytical ultracentrifuge would appear to have an important role to play in the battle against important kinase related diseases and in our understanding of the factors that underpin antimicrobial resistance [52,57].

## Figures and Tables

**Table 1:** Comparative hydrodynamic parameters for the intact band 3 protein, and its cytoplasmic (CD) and transmembane (TMD) domains examined separately.

	CD Domain	TMD Domain	Intact Band 3
Solvent:	pH8 Tris Buffer	C_12_E_8_	Reduced Triton
detergent binding ^1^	-	0.945 g/g	0.77 g/g
partial specific volumes ^2^: $\bar{v}\;$protein $\bar{v}\;$detergent $\bar{v}\;$complex	0.740 mL/g	0.7642 mL/g (0.9732 ± 0.0003) mL/g 0.866 mL/g	(0.740 ± 0.007) mL/g (0.9732 ± 0.0003) mL/g (0.842 ± 0.004) mL/g
monomer molecular mass *M*_1_ (Da)	40,000	122,800	200,000
molecular mass *M*_w_ in solution (Da)	80,000 (dimer)	250,000 (dimer)	400,000–800,000 (dimer-tetramer) + some hexamer/higher order associations
dissociation constant *K*_d_ (μM)	2.8 ± 0.5	<1	
sedimentation coefficient *s*^o^_20,w_ (S)	3.74 ± 0.07	4.94 ± 0.07	dimer = (6.9 ± 0.1) S tetramer = (10.6 ± 0.7) S
translational frictional ratio *f/f*_o_	1.7 ± 0.2	1.29 ± 0.02	dimer = (1.55 ± 0.080) tetramer = (1.68 ± 0.28)
Perrin *P* shape parameter (based on hydration = 0.2)	1.5	1.15 ± 0.05	*P*_dimer_ = 1.44 ± 0.08 *P*_tetramer_ = 1.49 ± 0.21
axial ratio *a/b*	~10	~3.5	dimer *a/b* ~7 tetramer *a/b* ~10

^1^ Essential for the correct evaluation of the molecular mass of the protein; ^2^ Essential for the correct evaluation of the bouyancy parameter (1- $\bar{v}$ ρ_o_) where ρ_o_ is the density of the solvent. Data from [1,10,11].

## Abbreviations

AUC: analytical ultracentrifugation; CD, cytoplasmic domain; SE, sedimentation equilibrium; SV, sedimentation velocity; TMD, transmembrane domain.
